# Tubal patency following surgical and clinical treatment of ectopic pregnancy

**DOI:** 10.1590/S1516-31802006000500005

**Published:** 2006-09-07

**Authors:** Julio Elito, Kyung Koo Han, Luiz Camano

**Keywords:** Ectopic pregnancy, Methotrexate, Beta-hCG, Hysterosalpingography, Chorionic gonadotropin, Infertility, Gravidez ectópica, Metotrexato, Histerossalpingografia, Gonadotropina coriônica, Infertilidade

## Abstract

**CONTEXT AND OBJECTIVE:**

As there is little information about fertility outcomes among women following clinical treatment (methotrexate and expectant management) and surgery (salpingectomy) consequent to ectopic pregnancy, we evaluate the results from hysterosalpingography subsequent to treatment. The objective was to evaluate contralateral tubal patency using hysterosalpingography following surgery and clinical treatment of tubal pregnancy.

**DESIGN AND SETTING:**

This was a prospective study at the Department of Obstetrics of Universidade Federal de São Paulo, a tertiary center.

**METHOD:**

Among 115 patients who underwent hysterosalpingography following surgery and clinical treatment of tubal pregnancy between April 1994 and February 2002, 30 were treated with a single intramuscular dose of methotrexate (50 mg/m^2^), 50 were followed up expectantly and 35 underwent salpingectomy.

**RESULTS:**

The patency of the ipsilateral tube was 84% after methotrexate treatment and 78% after expectant management. In addition, contralateral tubal patency was 97% after methotrexate treatment, 92% after expectant management and 83% after salpingectomy. There were no statistically significant differences between the clinical treatment and surgery groups.

**CONCLUSIONS:**

The findings from this study suggest similar contralateral tubal patency rates following salpingectomy, methotrexate treatment and expectant management.

## RESUMO

**CONTEXTO E OBJETIVO:**

Como existem poucas informações a respeito do futuro reprodutivo das pacientes tratadas de gravidez ectópica através do tratamento clínico (metotrexato e conduta expectante) e da cirurgia (salpingectomia), avaliamos as histerossalpingografias após o tratamento da gravidez ectópica. O objetivo foi avaliar a permeabilidade da tuba contralateral pela histerossalpingografia após o tratamento cirúrgico e clínico da gravidez tubária.

**TIPO DE ESTUDO E LOCAL:**

Estudo prospectivo realizado no Departamento de Obstetrícia da Universidade Federal de São Paulo (centro terciário).

**MÉTODOS:**

Foram realizadas 115 histerossalpingografias após o tratamento clínico e cirúrgico da gravidez tubária no período de 1994 a 2002, sendo que 30 após o tratamento com dose única de metotrexato (50 mg/m^2^) intramuscular, 50 após a conduta expectante e 35 após a salpingectomia.

**RESULTADOS:**

A permeabilidade tubária ipsilateral foi de 84% após o tratamento com metotrexato e de 78% após a conduta expectante. A permeabilidade da tuba contralateral foi de 97% após o tratamento com metotrexato, 92% após a conduta expectante e 83% após a salpingectomia. Não houve diferença estatisticamente significante entre os grupos de tratamento cirúrgico e clínico.

**CONCLUSÃO:**

Os dados deste estudo sugerem que a permeabilidade tubária contralateral é similar após a salpingectomia, o tratamento com metotrexato e a conduta expectante.

## INTRODUCTION

The incidence of ectopic pregnancy (EP) has been increasing recently.^1^ Diagnosis has improved through evaluation of the levels of beta-human choriongonadotropin hormone (beta-hCG) and through transvaginal ultrasound.^2^ Early detection makes it possible to perform conservative treatment, such as expectant management or medical treatment with methotrexate. In spite of the advances in diagnosis, in some latent cases, the diagnosis is delayed, and in most of them surgery to remove the tube is necessary. These patients' reproductive future depends on certain factors such as the patient's age, history of infertility, previous ectopic pregnancy, previous tubal rupture and involvement of the contralateral tube. The fertility outcome for these patients can be evaluated by hysterosalpingography or future pregnancy.^3,4^ Hysterosalpingography (HSG) is an important diagnostic method following treatment of ectopic pregnancy.^5,6^ If the results demonstrate obstruction in both tubes, spontaneous pregnancy is not possible.

As there is little information about fertility outcomes among women treated conservatively by either methotrexate or expectant management, in relation to patients undergoing surgery with salpingectomy, the results subsequent to HSG are presented here.

## OBJECTIVE

In this study, contralateral tubal patency was evaluated using HSG following surgery and clinical treatment of ectopic pregnancy.

## METHODS

### Patients

This descriptive study was conducted between April 1994 and February 2002. During this period, 380 cases of unruptured ectopic pregnancy were diagnosed: 60 of them underwent systemic treatment with methotrexate (50 mg/m^2^ intramuscularly), 200 were observed by means of expectant management and 120 cases underwent surgery. HSG was performed on 115 patients, who were divided into three groups: 30 treated with a single dose of methotrexate (MTX), 50 with expectant management and 35 with salpingectomy.

The study was performed at the Department of Obstetrics of Universidade Federal de São Paulo, which is a tertiary center. The study was approved by the Ethics Committee of the Institution. All patients who took part in the study had signed a written letter of consent in which the entire procedure was described.

### Diagnosis of ectopic pregnancy

Ectopic pregnancy was diagnosed by means of evaluation of the patient's previous clinical history, gynecological examination, transvaginal ultrasound and quantitative assaying of serum beta-hCG. In all the cases the diagnosis was confirmed from the ultrasound examination, by viewing an image characteristic of an extraovarian adnexal mass. Transvaginal ultrasound was carried out using a 7.5-MHz transvaginal probe, with Synerg equipment (General Electric, Milwaukee, United States). After the confirmation of the diagnosis, in cases where the adnexal mass was less than or equal to 3.5 cm, two beta-hCG titers were obtained at 24 to 48-hour intervals.

The serum beta-hCG concentration was measured using the enzyme immunoassay method (AIA-Pack beta-hCG, Tosoh Medic, Inc., Yamaguchi, Japan), in accordance with the Third International Standard.^7^

The criterion for ultrasound confirmation was the presence of an extraovarian adnexal mass in patients with suspected ectopic pregnancy (amenorrhea, bleeding and pain), with positive beta-hCG. The rule for performing the ultrasound was first to evaluate the cavity of the uterus in order to exclude entopic pregnancy (except for cases of heterotopic pregnancy). Next, the ovaries were evaluated and, after identifying them, the corpus luteum was located. Then, the vicinity of this ovary was searched to find an extraovarian adnexal mass (live embryo or tubal ring or hematosalpinx), since in most cases the ectopic pregnancy is on the same side as the corpus luteum. If an extraovarian adnexal mass was not seen on the same side as the corpus luteum, the other side would need to be searched. When the ultrasound examination was performed in this sequence, the diagnosis of ectopic pregnancy was confirmed in all cases in this study.

### Hysterosalpingography

The HSG was performed under fluoroscopic observation using a medium-sized water-soluble catheter. An abnormality was recorded if dye was not seen to spill from a distal tubal site. The equipment was a Legend CR9 (General Electrics, Hungary).

### Treatments

#### Methotrexate

The inclusion criteria^8^ for MTX treatment were: hemodynamic stability, increased or maintained beta-hCG levels at 24 and 48-hour intervals, cross-sectional diameter of the mass ≤ 3.5 cm, desire for future pregnancy and consent to participate in the study through a document signed and approved by the Ethics Committee of the Institution. Patients who were clinically unstable or who had a history of sensitivity to MTX or who suffered from blood dyscrasia or liver and kidney diseases were excluded.

The patients who satisfied the selection criteria were treated with a single intramuscular dose of MTX (50 mg/m^2^). As follow-ups, beta-hCG levels were ascertained on days one, four and seven after MTX injection. The protocol stipulated that any patient who did not have a decline of 15% in beta-hCG levels between days four and seven would be given a second dose of MTX (50 mg/m^2^ intramuscularly) one week after the first dose. Patients with declining beta-hCG levels between days four and seven were monitored as outpatients weekly until their beta-hCG levels were below 5 mIU/ml. Hospitalization was indicated in cases with significant abdominal pain, or suspected tubal rupture. The treatment was considered to be a success when the levels of beta-hCG became negative (< 5 mUI/ml), and to be a failure when surgery was necessary.

HSG was performed **only on patients whose clinical treatment was successful.** The examination was performed after three or six months, at the end of the treatment, because the adnexal mass usually disappeared from ultrasound images during this period, or when the patient decided to try for a new pregnancy.

#### Expectant management

The inclusion criteria for expectant management were: hemodynamic stability, decline of beta-hCG levels at 24 and 48-hour intervals, beta-hCG level < 3,000 mIU/ml, cross-sectional diameter of the mass ≤ 3.5 cm, desire for future pregnancy and consent to participate in the study, through a document signed and approved by the Ethics Committee of the Institution.

The patients were monitored weekly until the levels of beta-hCG were below 5 mIU/ml. HSG was performed **only on patients whose the expectant management was successful.** The examination was performed after the tubal mass disappeared from ultrasound images, or when the patient decided to try for a new pregnancy.

#### Surgery

The inclusion criteria for surgery (salpingectomy) were: hemodynamic instability, tubal rupture, failure of clinical treatment, cross-sectional diameter of the mass > 3.5 cm, not desiring a future pregnancy and failure of conservative surgery (salpingostomy). HSG was performed eight weeks after the surgery

**or when the patient decided to try for a new pregnancy.**

#### Statistical analysis

To compare the post-treatment tubal patency levels seen via HSG in the contralateral tube following MTX, expectant management and salpingectomy, the Fisher exact test with 5% significance level was applied.

## RESULTS

Free passage through the ipsilateral tube after MTX treatment was seen in 84% of the cases (25 out of 30). After expectant management, it was seen in 78% of the cases (39 out of 50). The contralateral tube patency levels were 97%, 92% and 83%, following methotrexate treatment, expectant management and salpingectomy, respectively (Table 1). There was no significant difference between the groups.

**Table 1:** Post-treatment tubal patency seen via hysterosalpingography on the contralateral tube following methotrexate (MTX), expectant management and surgery

Hysterosalpingography	MTX n (%)	Expectant management n (%)	Salpingectomy n (%)	Total n (%)
Obstruction	2 (3%)	4 (8%)	10 (25%)	**11 (9%)**
Patency	30 (97%)	46 (92%)	30 (75%)	**104 (91%)**
**Total**	**32 (100%)**	**50 (100%)**	**40 (100%)**	**115 (100%)**

p = 0.1487.

## DISCUSSION

Fertility can be evaluated indirectly via HSG and directly by pregnancy. HSG is considered to be a good examination for evaluating the tubal patency, in spite of the inconveniences and doubts about the interpretation of the exam.^9,10^

The HSG findings from the ipsilateral tube showed that 84% (MTX) and 78% (expectant management) of the diseased tubes were open, which is similar to the rates in other reports.^3,4,6^ The patency of the contralateral tube was 97% (MTX), 92% (expectant management) and 83% (salpingectomy), which is also similar to the rates in other reports.^6,11,12^

Obstruction of the ipsilateral tube occurred in 16% of the MTX group and 22% of the expectant management group. Comparing these results with obstruction of the contralateral tube (3% MTX and 8% expectant management) showed that the difference between the obstruction rates for the ipsilateral and contralateral tubes could be post-treatment sequelae of the tubal pregnancy.

Thus, the contralateral tube probably gives a picture of the tube disease prior to the tubal pregnancy. The difference between obstruction of the ipsilateral and contralateral tubes following clinical treatment may demonstrate the cases that suffer from sequelae of non-surgical treatment.^7,11-13^

We believe that this spontaneous healing of ectopic pregnancy in selected cases without any tubal interventions should not harm the tube and should result in a good long-term fertility outcome. However, normal radiological findings show nothing regarding tubal function, since a disturbance in the tube may also be a cause of ectopic pregnancy. On the other hand, if the HSG demonstrates obstruction of the tubes, these results reduce the possibility of a spontaneous pregnancy and these patients should be treated with in vitro fertilization to conceive.

Obstruction of the contralateral tube contributes important information about the case. This happened in 3% of the MTX cases and in 8% of the expectant management cases. These results suggest that, before developing the ectopic pregnancy, some patients had an obstruction in the contralateral tube, probably caused by salpingitis. In the group that underwent surgery (salpingectomy), contralateral tubal obstruction occurred in 17% of the cases. Obstruction of the contralateral tube was more frequent in the group that underwent surgery than in the clinical treatment groups. The explanation for these results is probably that surgery may give rise to more occurrences of adhesions because of the peritoneal factor, thereby resulting in infertility. However, in the present study there was no significant difference between the groups in relation to contralateral tubal patency.

It is important to mention that the three groups in this study (expectant management, methotrexate and salpingectomy) basically differed by definition. Statistical analysis on the sample for this study was unable to identify any significant difference, precisely because of the small sample size.

Although the observed difference did not reach the usual significance level, it should be noted that the data favor clinical treatment. It would be wise for more patients to be studied before accepting the equivalence between the treatments as a definite finding.

## CONCLUSIONS

Thus, no significant difference in patency of the contralateral tube was demonstrated between the groups. These results show that clinical treatment (MTX or expectant management) and surgery (salpingectomy) do not affect the tubal patency differently.
